# FGFR1 clustering with engineered tetravalent antibody improves the efficiency and modifies the mechanism of receptor internalization

**DOI:** 10.1002/1878-0261.12740

**Published:** 2020-07-03

**Authors:** Marta Pozniak, Aleksandra Sokolowska‐Wedzina, Kamil Jastrzebski, Jakub Szymczyk, Natalia Porebska, Mateusz Adam Krzyscik, Malgorzata Zakrzewska, Marta Miaczynska, Jacek Otlewski, Lukasz Opalinski

**Affiliations:** ^1^ Faculty of Biotechnology Department of Protein Engineering University of Wroclaw Poland; ^2^ Laboratory of Cell Biology International Institute of Molecular and Cell Biology Poland; ^3^ Faculty of Biotechnology Department of Protein Biotechnology University of Wroclaw Poland

**Keywords:** CIE, CME, endocytosis, FGFR, protein transport, signaling

## Abstract

Fibroblast growth factor receptor 1 (FGFR1) transmits signals through the plasma membrane regulating essential cellular processes like division, motility, metabolism, and death. Overexpression of FGFR1 is observed in numerous tumors and thus constitutes an attractive molecular target for selective cancer treatment. Targeted anti‐cancer therapies aim for the precise delivery of drugs into cancer cells, sparing the healthy ones and thus limiting unwanted side effects. One of the key steps in targeted drug delivery is receptor‐mediated endocytosis. Here, we show that the efficiency and the mechanism of FGFR1 internalization are governed by the spatial distribution of the receptor in the plasma membrane. Using engineered antibodies of different valency, we demonstrate that dimerization of FGFR1 with bivalent antibody triggers clathrin‐mediated endocytosis (CME) of the receptor. Clustering of FGFR1 into larger oligomers with tetravalent antibody stimulates fast and highly efficient uptake of the receptor that occurs via two distinct mechanisms: CME and dynamin‐dependent clathrin‐independent endocytic routes. Furthermore, we show that all endocytic pathways engaged in FGFR1 internalization do not require receptor activation. Our data provide novel insights into the mechanisms of intracellular trafficking of FGFR1 and constitute guidelines for development of highly internalizing antibody‐based drug carriers for targeted therapy of FGFR1‐overproducing cancers.

## Graphical abstract

FGFRs are overproduced by cancer cells and constitute attractive molecular target for therapy using antibody–drug conjugates (ADCs). The endocytosis of FGFRs is a critical step of ADC approach. Using engineered antibodies of different valency, we demonstrate that the efficiency and the mechanism of FGFR1 endocytosis are dictated by the spatial distribution of the receptor in the plasma membrane.

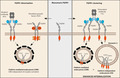

**Abbreviations**
ADCs: antibody–drug conjugates
AP2: adaptin 2 complex
B‐Fc: bivalent anti‐FGFR1 antibody
BLI: biolayer interferometry
BN‐PAGE: blue native polyacrylamide gel electrophoresis
CH2: heavy chain constant domain 2
CH3: heavy chain constant domain 3
CHC: clathrin heavy chain
CIE: clathrin‐independent endocytosis
CLIC: clathrin‐independent carriers pathway
CME: clathrin‐mediated endocytosis
DLS: dynamic light scattering
DNM2: dynamin‐2
EEA1: early endosome antigen 1
ERK1/2: extracellular signal‐regulated kinase ½
Fc: fragment crystallizable region
FGF: fibroblast growth factor
FGFR: fibroblast growth factor receptor
IgG1: immunoglobulin G1
*K*_D_: equilibrium dissociation constant
*k*_off_: dissociation constant
*k*_on_: association constant
PDGFR: platelet‐derived growth factor receptor
RTK: receptor tyrosine kinase
scFv: single chain variable fragment
SPR: surface plasmon resonance
T‐Fc: tetravalent anti‐FGFR1 antibody
VEGFR: vascular endothelial growth factor receptor

## 1 Introduction

Fibroblast growth factor receptor 1 (FGFR1) is a receptor tyrosine kinase (RTK) that, when activated by extracellular ligands, fibroblast growth factors (FGFs), transduces signals through the plasma membrane [1,2]. FGFR1 is composed of an extracellular region involved in FGF binding, a single transmembrane span and an intracellular tyrosine kinase domain [1,2]. The extracellular domain of FGFR1 contains three immunoglobulin‐like domains D1, D2, and D3. The D1 domain fulfills a regulatory function preventing FGFR1 from autoactivation in the absence of FGFs [3, 4, 5, 6]. The D2 and D3 domains form FGF binding sites [1]. Additionally, the extracellular region of FGFR1 includes binding site for heparans and the region enriched in acidic residues, so‐called acidic box [3]. The transmembrane domain embeds FGFR1 in the plasma membrane and participates in the receptor dimerization [2]. The intracellular region of FGFR1 includes the juxtamembrane region of regulatory function and a split tyrosine kinase directly involved in signal transmission [2]. FGFR1‐dependent signaling controls pivotal cellular processes like cell division, migration, metabolism, and apoptosis [1,2,7]. The elevated levels of FGFR1 were found in numerous tumors, including breast, lung, head, and neck cancers, and are predictors of poor outcome in patients [8, 9, 10, 11, 12, 13, 14, 15, 16]. Therefore, FGFR1 is an attractive molecular target for selective cancer treatment.

One of emerging targeted anti‐cancer therapies is antibody–drug conjugates (ADCs) [17, 18, 19]. Typically, ADCs are composed of a monoclonal antibody that is linked to a highly cytotoxic drug by a specific linker. The monoclonal antibody provides the specificity of ADC, as it selectively delivers the cytotoxic payload via receptor‐mediated endocytosis to lysosomes of the cancer cells [17]. The specific linker sequence constitutes the cleavage site for lysosomal proteases. Once in lysosomes the proteinaceous part of ADCs is proteolytically degraded and cytotoxic drug, capable of crossing cellular membranes and reaching the intracellular targets is released [17]. Up to date, several ADCs have been approved and are commercially available for treatment of various cancers [18]. Importantly, a number of selective cytotoxic conjugates including ADCs against cancers overproducing FGFRs were generated,however, their *in vivo* therapeutic potential awaits further evaluation [20, 21, 22, 23, 24, 25, 26, 27]. A critical step in the anti‐cancer therapy with ADCs is selective and efficient delivery of the cytotoxic drug to the cell interior [28]. For this purpose, ADCs utilize endocytosis of the cancer‐specific ADC receptor [28]. Therefore, the knowledge about cellular mechanisms responsible for the uptake of cancer‐specific cell surface receptors is critical for ADC strategy.

The mechanisms involved in FGFR1 internalization are only partially understood [29]. It was demonstrated that FGFR1 is subjected to constitutive, low‐rate internalization, however, FGFR1 dimerization caused by ligand binding largely accelerates uptake of the receptor [30, 31, 32, 33]. The efficiency and mechanism of FGFR1 internalization depend on the type of an applied ligand [29,34, 35, 36, 37, 38, 39]. The uptake of FGF/FGFR1 complexes mainly occurs via clathrin‐mediated endocytosis (CME) leading to lysosomal degradation of FGFR1 [29,30,36,37,39]. We have recently uncoupled FGFR1 dimerization from the receptor activation and have demonstrated that dimerization of FGFR1, but not receptor autophosphorylation, triggers CME of FGFR1 [5,6]. A number of proteins involved in CME of FGFR1 in response to FGF binding were identified,however, the exact role of a number of these factors in the receptor internalization is still largely mysterious [31,40, 41, 42, 43, 44]. Besides CME, clathrin‐independent endocytosis (CIE) may participate in FGFR1 internalization [34,45,46]. A mechanism determining which particular endocytic route(s) will be employed by FGFR1 is currently unknown.

Here, we demonstrate that the efficiency and the mechanism of FGFR1 uptake are dictated by the oligomeric state of the receptor in the plasma membrane. Using engineered anti‐FGFR1 antibody fragments of different valency as tools for differential FGFR1 clustering, we demonstrate that bivalent antibodies stimulate CME of FGFR1, while the cell entry of tetravalent antibody‐FGFR1 complexes is split between two distinct endocytic pathways: CME and CIE that requires dynamin‐2. The switch in an endocytic mechanism is associated with largely improved efficiency of FGFR1 internalization and receptor degradation. Importantly, our data show that both CME and CIE of FGFR1 do not require activation of the receptor.

## 2 Methods

### 2.1 Antibodies and reagents

The primary antibodies directed against FGFR1 (#9740), phospho‐FGFR (pFGFR; #3476), ERK1/2 (#9102), and phospho‐ERK1/2 (pERK1/2; #9101) were from Cell Signaling (Danvers, MA, USA). Anti‐tubulin primary antibody (#T6557), anti‐GST antibody (#G1160), and anti‐beta actin antibody (#A5441) were from Sigma‐Aldrich (St Louis, MO, USA). Anti‐clathrin heavy chain (#610499), anti‐dynamin (#610245), and anti‐AP2µ2 primary antibodies were from BD Biosciences (Franklin Lakes, NJ, USA). Anti‐galectin‐3 (#sc‐20157), anti‐ROCK1 (#sc17794), anti‐ROCK2 (#sc398519), and anti‐CD44 (#sc‐7297) primary antibodies were from Santa Cruz Biotechnology (Dallas, TX, USA). Anti‐human IgG (Fc) antibody coupled to HRP (# 4‐10‐ 20) was from KPL (Gaithersburg, MA, USA). Secondary antibodies coupled to HRP were from Jackson Immuno‐Research Laboratories (Cambridge, UK). Anti‐PDGFRβ antibody was from R&D Systems (Minneapolis, MN, USA), and anti‐VEGFR2 antibody was from Thermo Fischer Scientific (Waltham, MA, USA). Anti‐EEA1 primary antibody (ALX‐210‐239) was from Enzo Life Sciences (Farmingdale, NY, USA). Donkey anti‐rabbit AF‐647 secondary antibody used for EEA1 immunofluorescence was from Thermo Fisher Scientific.

Protein A Sepharose and Glutathione Sepharose resins were from GE Healthcare (Piscataway, NJ, USA). siRNA was from Thermo Fisher Scientific or from GE Dharmacon (Lafayette, CO, USA) as described previously [47].

### 2.2 Cells

CHO‐S cells (Thermo Fisher Scientific) were cultured in protein‐ and serum‐free PowerCHO‐2CD medium (Lonza, Alpharetta, GA, USA) supplemented with antibiotic mix (100 U·mL^−1^ penicillin and 0.1 mg·mL^−1^ streptomycin) (Thermo Fisher Scientific) and 8 mm l‐glutamine (Thermo Fisher Scientific). Cells were grown at 37 °C with 8% CO_2_ in a shaking incubator (140 rpm). Routine subculturing was carried out every 2–3 days at seeding density of 0.2–0.3 × 10^6^ cells·mL^−1^.

Human osteosarcoma cell line (U2OS) was obtained from American Type Culture Collection (ATCC), and U2OS cells stably expressing FGFR1 (U2OS‐R1) were a kind gift from Dr. E.M. Haugsten from the Norwegian Radium Hospital. Both cell lines were cultured in 5% CO_2_ atmosphere at 37 °C in Dulbecco’s Modified Eagle’s Medium (Biowest, Nuaille, France) supplemented with 10% fetal bovine serum (Thermo Fisher Scientific) and antibiotic mix (100 U·mL^−1^ penicillin and 100 μg·mL^−1^ streptomycin) (Thermo Fisher Scientific). In the case of U2OS‐R1 cells, medium contained also 1 mg·mL^−1^ geneticin (Thermo Fisher Scientific). Cells were grown in 5% CO_2_ atmosphere at 37 °C. Cells were seeded into tissue culture plates one day prior to the start of the experiments. Murine embryonic fibroblasts (NIH3T3) were from ATCC and were cultured in Dulbecco’s Modified Eagle’s Medium (Biowest) supplemented with 2% bovine serum (Thermo Fisher Scientific) and antibiotic mix (100 U·mL^−1^ penicillin and 100 μg·mL^−1^ streptomycin) (Thermo Fisher Scientific). Cells were grown in 5% CO_2_ atmosphere at 37 °C. Cells were seeded into tissue culture plates one day prior to the start of the experiments.

Transfections of siRNA were performed with Lipofectamine RNAiMAX Transfection Reagent (Thermo Fisher Scientific) according to the manufacturer’s instructions.

### 2.3 siRNA transfection

Cells were transfected with siRNA against endocytic proteins with Lipofectamine RNAiMAX (Thermo Fisher Scientific), as described previously [47]. U2OS‐R1 cells were treated for 3 days in 5% CO_2_ atmosphere at 37 °C with 20 nm Ambion Silencer Select siRNA against clathrin heavy chain (CHC_1 ‐ #s475, CHC_2‐ #s477), a μ2 subunit of the AP2 complex (AP2μ2) (AP2M1_1 ‐ #s3112, AP2M1_2 ‐ #s3113), dynamin‐2 (DNM2_1 ‐ #s4212, DNM2_2 ‐ #s4213), galectin‐3 (GAL3_1 ‐ #s8148, GAL3_2‐ #s8149), CD44 (CD44_1‐ #s2681), ROCK1 (ROCK1_1 ‐ #s12097, ROCK1_2 ‐#s12098), and ROCK2 (ROCK2_1 ‐ #s18161, ROCK2_2‐ #s18162) were from Thermo Fisher Scientific. For double‐depletion, selected siRNA against endocytic proteins was mixed with each other (at the concentration of 20 nm each) or Ctrl_1 siRNA (20 nm) to equalize the amount of siRNA used. Control cells were transfected with Ctrl_1 alone (40 nm).

### 2.4 Recombinant proteins

Fully glycosylated extracellular domains of FGFRs fused to the Fc fragment of human IgG1: FGFR1 IIIc (FGFR1‐Fc), FGFR2 IIIc (FGFR2‐Fc), FGFR3 IIIc (FGFR3‐Fc), and FGFR4 (FGFR4‐Fc), and the extracellular part of FGFR1 lacking the N‐terminal D1 domain (FGFR1 D2‐D3) were produced as described previously by our group [48]. FGFR1 GST‐D1 was expressed in *E. coli* BL21 CodonPlus (DE3)‐RIL (Agilent Technologies, Santa Clara, CA, USA) and purified with use of Glutathione Sepharose column as described previously [5,6,49].

T‐Fc was constructed based on the B‐Fc by the in frame fusion of second anti‐FGFR1 scFv coding sequence [25]. The scFv proteins in the Fc format (scFv‐Fc) were expressed in CHO‐S cells. One day before the transfection, cells were seeded at a density of 1.8 × 10^6^ cells·mL^−1^ in culture medium. On the transfection day, CHO‐S cells were centrifuged and cell pellet was re‐suspended in protein‐ and serum‐free ProCHO4 medium at 2 × 10^6^ cells·mL^−1^. Plasmids encoding T‐Fc or B‐Fc (1.25 µg DNA per 1 × 10^6^ cells) and PEI (5 µg per 1 × 10^6^ cells) were diluted separately in 150 mm NaCl, mixed, and incubated at RT for 10 min. After this time, the solution was added to the cell culture. Cells were incubated under standard conditions for 4 h (37 °C, 140 rpm, 8% CO_2_). After this time, the cell culture was diluted with an equal volume of PowerCHO‐2CD supplemented with 4 mm L‐glutamine and antibiotic mix (200 U·mL^−1^ penicillin and 200 μg·mL^−1^ streptomycin) to obtain the cell density of about 1 × 10^6^ cells·mL^−1^, and incubated at 32 °C. Next day, the cell culture was supplemented with 8 mm L‐glutamine and finally harvested at day 12. B‐Fc and T‐Fc antibodies were purified on HiTrap MabSelect column. Both proteins were eluted with 0.1 m sodium citrate, pH 3.5, and neutralized with 1 m Tris‐HCl, pH 9.0. The identity and the purity of the proteins were confirmed by MALDI‐MS, SDS/PAGE, and western blotting. Recombinant PDGFRβ and VEGFR2 were from R&D Systems. Recombinant galectin‐1, galectin‐3, and FGF1 were obtained as described previously [31,50].

### 2.5 SPR and BLI measurements

SPR experiments were performed on the Biacore 3000 instrument (GE Healthcare) at 25 °C. For selectivity analysis of B‐Fc and T‐Fc against FGFR1, CM5 sensors were coated with FGFR1‐Fc (at 825 RU), PDGFRβ (at 825 RU), and VEGFR2 (at 825 RU). T‐Fc, B‐Fc (1 μm), and anti‐VEGFR2 or anti‐PDGFRβ antibodies (controls) were injected independently over all sensors at 30 μL·min^−1^ flow rate, and the association and dissociation were monitored for 240 s. For specificity analysis of engineered antibodies for FGFR types, FGFR1‐Fc (at 825 RU), FGFR2‐Fc (at 1000 RU), FGFR3‐Fc (at 1000 RU), and FGFR4‐Fc (at 1000 RU) were immobilized on CM5 sensors. Next, FGF1 (control), T‐Fc, or B‐Fc (1 μm) were injected independently over all sensors at 30 μL·min^−1^ flow rate and the association and dissociation were monitored for 240 s. For analysis of epitopes for both antibodies within FGFR1, CM5 sensors were coated with FGFR1.D1‐D2‐D3‐Fc (at 825 RU), FGFR1.D2‐D3‐Fc (at 900 RU), and GST‐tagged D1 domain of FGFR1 (GST‐D1) (at 300 RU). Next, proteins (1 μm) were injected independently over all sensors at 30 μL·min^−1^ flow rate and the association and dissociation were monitored for 240 s. Measurements were performed in PBS‐PN buffer (0.005% v/v surfactant P20, 0.02% NaN_3_ in PBS; pH 7.2), and chip surface was regenerated with 10 mm glycine, pH 1.5. All sensograms were analyzed using BIAevaluation 4.1 software (GE Healthcare).

To determine the kinetic parameters of antibodies–FGFR1 interaction, FGFR1‐Fc was immobilized at 1000 RU on the CM4 sensor. Various concentrations of engineered antibodies (0.625–20 nm) were applied on the sensor and measured for 300 s (120 s for association and 180 s for dissociation) at 30 μL·min^−1^ flow rate. Measurements were performed in PBS with 0.05% Tween 20, 0.02% NaN_3_, pH 7.2, and chip surface was regenerated with 10 mm glycine, pH 1.5. Kinetic constants (*k*_on_, *k*_off_, and *K*_D_) were calculated using BIAevaluation 4.1 software using 1 : 1 Langmuir binding model with drifting baseline. BLI was performed using Octet RED K2 system (ForteBio, San Jose, CA, USA). To analyze differences in the FGFR1 binding between B‐Fc and T‐Fc, FGFR1‐Fc (30 μg·mL^−1^) was immobilized on AR2G biosensors and incubated with B‐Fc (0.3 μm) and T‐Fc (0.3 μm). Measurements were performed in PBS buffer.

To analyze the impact of B‐Fc and T‐Fc on the FGFR1 interaction with partner proteins, the competitive BLI was applied. FGFR1‐Fc (30 μg·mL^−1^) was immobilized on ProtA biosensors, and sensors were either left untreated or incubated with saturating concentrations of engineered antibodies. Subsequently, the binding of FGFR1 partner proteins, FGF1 (50 μg·mL^−1^), galectin‐1 (50 μg·mL^−1^), or galectin‐3 (50 μg·mL^−1^), to the receptor was measured in the presence or absence of B‐Fc or T‐Fc (0.3 μm). Measurements were performed in PBS buffer.

### 2.6 Interaction of engineered antibodies with cells

U2OS and U2OS‐R1 cells were briefly incubated at RT with engineered antibodies (100 nm), washed, and lysed. Cell‐bound B‐Fc and T‐Fc were detected with western blotting using anti‐Fc antibodies. The quantification of signals was performed with image lab software (Bio‐Rad, Hercules, CA, USA) from three independent experiments. The statistical significance was assessed using *t*‐test; *P < 0.05, **P < 0.005, n.s.—not significant.

### 2.7 Dynamic light scattering

Dynamic light scattering (DLS) experiments were performed with FGFR1‐Fc (0.2 mg·mL^−1^), T‐Fc (0.2 mg·mL^−1^) and mix of the both proteins (FGFR1‐Fc (0.15 mg·mL^−1^) and B‐Fc (0.3 mg·mL^−1^)) in PBS. The measurements were executed using a DynaPro^®^ NanoStar^®^ (Wyatt Technology, Santa Barbara, CA, USA) equipped with a 658 nm (red) laser. Analyses were performed in disposable MicroCuvettes (Wyatt Technology) at 20 °C, and each measurement consisted of ten, 5‐second runs. The DLS data were collected and analyzed using dynamics v7 software (Wyatt Technology). All DLS‐based hydrodynamic diameters and molecular mass were determined by cumulants analysis using Rayleigh spheres model.

### 2.8 Blue Native PAGE

Blue native PAGE (BN‐PAGE) experiments were performed with B‐Fc (0.2 μm and 1 μm), T‐Fc (0.2 μm and 1 μm), and their mixtures with FGFR1‐Fc (0.1 μm) or FGFR1 D1‐GST (0.165 μm) at RT for 10 min in PBS buffer. Proteins were separated using 4–10% BN‐PAGE gradient gels and subjected to western blotting using anti‐FGFR1 or anti‐GST antibodies.

### 2.9 FGFR1 activation and downstream signaling cascades

To analyze the impact of T‐Fc and B‐Fc on the FGFR1 activation and initiation of receptor‐downstream signaling cascades, serum‐starved NIH3T3 cells were incubated for 15 min with B‐Fc (13 nm, 65 nm), T‐Fc (13 nm, 65 nm), and FGF1 (50 ng·mL^−1^) in the presence of heparin (10 U·mL^−1^). Cells were lysed in Laemmli buffer and subjected to SDS/PAGE and western blotting.

To study the influence of engineered antibodies on the FGF1‐dependent activation of FGFR1, serum‐starved NIH3T3 cells were stimulated with FGF1 (50 ng·mL^−1^) for 15 min in the absence or in the presence of B‐Fc (13 nm, 65 nm) or T‐Fc (13 nm, 65 nm) and heparin (10 U·mL^−1^). Cells were lysed in Laemmli buffer and subjected to SDS/PAGE and western blotting. Intensities of pFGFR, pERK1/2, and tubulin signals were quantified using image lab software from at least three independent experiments. The values obtained for pFGFR and pERK1/2 were normalized for differences in loading (tubulin signal). The statistical significance of results was analyzed with the t‐test.

### 2.10 FGFR1 degradation

For analysis of the kinetics of FGFR1 degradation, serum‐starved U2OS‐R1 cells were pretreated with cycloheximide (10 μg·mL^−1^), FGF1 (100 ng·mL^−1^, heparin 10 U·mL^−1^), B‐Fc (65 nm), or T‐Fc (65 nm) for up to 180 min in 5% CO_2_ atmosphere at 37 °C. At various time points, cells were lysed in Laemmli buffer and subjected to SDS/PAGE and western blotting. The quantitative analysis of FGFR1 levels was performed with image lab software from four independent experiments. The statistical significance of results was analyzed with the *t*‐test.

### 2.11 Fluorescence microscopy

#### 2.11.1 Wide‐field fluorescence microscopy

Binding analyses of B‐Fc and T‐Fc to the full‐length FGF1 were performed using U2OS‐R1 cells incubated with B‐Fc (100 nm) and T‐Fc (100 nm) for 15 min on ice to allow the formation of FGFR1–antibody complexes with simultaneous inhibition of the endocytosis. Alternatively, engineered antibodies were incubated briefly with cells at room temperature prior to fixing. Cells were washed in PBS and fixed in 4% paraformaldehyde, and B‐Fc or T‐Fc were visualized with Zenon AF‐488 (Thermo Fisher Scientific). The quantification of Zenon AF‐488 signal was done using zen 2.3 software (Zeiss, Oberkochen, Germany). For the analysis of FGFR1 dependence of the cellular uptake of engineered antibodies, U2OS‐R1 and U2OS cells were incubated with B‐Fc (100 nm) and T‐Fc (100 nm) for 30 min in 5% CO_2_ atmosphere at 37 °C. The internalization was stopped by cooling down the cells on ice. Next, cells were then fixed with 4% paraformaldehyde and permeabilized with PBS buffer supplemented with 0.1% Triton. B‐Fc and T‐Fc were fluorescently labeled with Zenon AF‐488. Wide‐field fluorescence microscopy was carried out using Zeiss Axio Observer Z1 fluorescence microscope (Zeiss). Images were taken using LD‐Plan‐Neofluar 40×/0.6 Korr M27 objective and Axiocam 503 camera (Zeiss). Zenon AF‐488 signal was visualized with a 450/490 nm bandpass excitation filter and a 550/590 nm bandpass emission filter. NucBlue Live signal was visualized with a 335/383 nm bandpass excitation filter and a 420/470 nm emission filter. Image analysis was carried out using zen 2.3 and imagej (NIH, Betheseda, MD, USA).

#### 2.11.2 Confocal microscopy

The intracellular co‐localization of engineered antibodies with an early endosome marker protein, EEA1, was analyzed with immunofluorescence protocol. U20S‐R1 cells were seeded on µClear 96‐well plates from Greiner Bio‐One (Kremsmunster, Austria) (#655096) and reverse transfected with siRNA. After 72 h, U2OS‐R1 cells were incubated either with B‐Fc or T‐Fc (both at 100 nm) for 30 min at 37 °C. Following stimulation, cells were transferred to ice and washed twice with ice‐cold PBS and ice‐cold 3% paraformaldehyde was added to cells for 15 min at RT. Then, cells were washed three times with PBS and processed directly with an immunofluorescence protocol or stored at 4 °C. Cells were incubated for 10 min with 0.1% (w/v) saponin (Sigma‐Aldrich), 0.2% (w/v) fish gelatin (Sigma‐Aldrich), and 5 mg·mL^−1^ BSA (BioShop) in PBS. Afterward, cells were incubated with appropriate primary antibodies diluted in 0.01% (w/v) saponin and 0.2% fish gelatin in PBS. Cells were incubated with primary antibodies (EEA1), and B‐Fc and T‐Fc proteins were fluorescently labeled with Zenon AF‐488 for 1 h and washed twice with 0.01% (w/v) saponin and 0.2% fish gelatin in PBS. Next, cells were incubated for 30 min with secondary antibodies and DAPI (Sigma‐Aldrich). The images were obtained using Opera Phenix confocal microscope (Perkin Elmer, Waltham, MA, USA) with 40×/1.1 water immersion objective. harmony software (version 4.8; Perkin Elmer) was used for image acquisition and analysis. At least twenty 16‐bit images with resolution 1024 × 1024 pixels and binning 2 were acquired per experimental condition. Flat‐filed correction (Advance algorithm from harmony software) was applied to all images before analysis. The image analysis was performed using spot detection function to detect mask for vesicles positive for Zenon AF‐488 or EEA1. Subsequently, the integral fluorescence of a particular marker from mask was counted. Cell number was determined by nuclei detection using DAPI signal. All data were normalized to cell number. Pictures were assembled in Photoshop (Adobe) with only linear adjustments of contrast and brightness.

To analyze the kinetics of T‐Fc and B‐Fc uptake, U2OS‐R1 cells were incubated with B‐Fc or T‐Fc (100 nm) at 37 °C. The internalization was stopped at different time points by cooling down the cells on ice. Cells were fixed, permeabilized, and engineered antibodies or EEA1 were visualized as described above. The quantitative analyses of engineered antibodies’ internalization were performed using harmony software (Perkin Elmer).

To analyze the contribution of distinct endocytic pathways to the uptake of T‐Fc and B‐Fc, siRNA‐mediated knockdown of endocytic proteins was applied in conjunction with high‐content quantitative confocal microscopy. Next, cells were incubated with B‐Fc or T‐Fc (100 nm) and fluorescently labeled transferrin as an internal control up to 60 min at 37 °C. Engineered antibodies and EEA1 were visualized as described above. The quantification of B‐Fc and T‐Fc internalization upon inhibition of distinct endocytic pathways was performed using harmony software (Perkin Elmer).

## 3 Results

### 3.1 Engineered antibody fragments of various valency bind the extracellular region of FGFR1 with high affinity and specificity

**Fig. 1:**
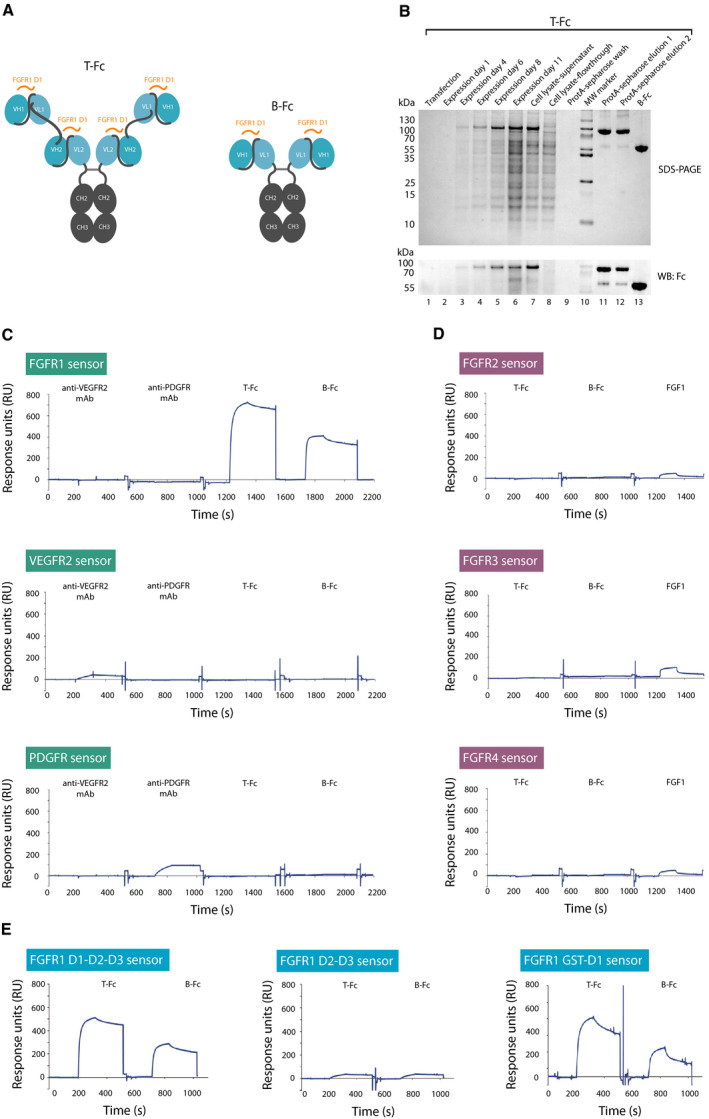
Characterization of anti‐FGFR1 engineered antibodies of different valency. (A) The schematic representation of structures of tetravalent (T‐Fc) and bivalent (B‐Fc) anti‐FGFR1 engineered antibodies. Fc region of IgG (CH2 and CH3 domains) is labeled in gray, and scFv proteins (VH and VL fusions) are marked in blue. Antibody regions recognizing epitopes within FGFR1 are marked in orange. (B) Expression and purification of T‐Fc and B‐Fc. Levels and purity of T‐Fc at different stages of protein expression and purification process were monitored with SDS/PAGE and western blotting with antibodies recognizing Fc fragment. (C) B‐Fc and T‐Fc are specific toward FGFR1. The extracellular regions of FGFR1, VEGFR2, and PDGFR were immobilized on SPR sensors and tested for the interaction with B‐Fc, T‐Fc, and commercial anti‐VEGFR2 and anti‐PDGFR antibodies with SPR. (D) Selectivity tests of B‐Fc and T‐Fc against FGFRs. The extracellular regions of FGFR2, FGFR3, and FGFR4 were immobilized on SPR sensors and tested for the interaction with B‐Fc, T‐Fc, and FGF1 as a control using SPR. (E) Engineered antibodies bind the D1 domain of the receptor. The full‐length extracellular domain of FGFR1 (D1‐D2‐D3), FGFR1 variant lacking the D1 domain (FGFR1 D2‐D3), and recombinant D1 domain (FGFR1 GST‐D1) were immobilized on SPR sensors and tested for interaction with B‐Fc and T‐Fc using SPR.

We have recently reported a phage display‐based selection of high‐affinity antibody fragments that selectively recognize epitopes within the D1 domain of the FGFR1 [25]. Furthermore, we have demonstrated that the bivalency and the high affinity promote internalization of FGFR1/engineered antibody complexes [5,6,49]. To study the impact of FGFR1 clustering into larger oligomeric structures on the receptor activity and intracellular trafficking, we generated tetravalent engineered antibody, T‐Fc, composed of two anti‐FGFR1 scFv fragments recognizing D1 domain of the receptor fused to the Fc fragment of human IgG1. T‐Fc assembles into tetravalent anti‐FGFR1 protein due to the interaction between CH2 and CH3 domains of the Fc region (Fig. 1A). Additionally, in our study we used previously reported bivalent anti‐FGFR1 engineered antibody, B‐Fc, composed of single scFv fused with the Fc (Fig. 1A) [25]. Both proteins were efficiently overproduced in CHO cells and purified using affinity chromatography (Fig. 1B). The purity and the identity of recombinant proteins were confirmed with western blotting and mass spectrometry (Fig. 1B, Fig. S1).

To study whether oligomerized scFv proteins in B‐Fc and T‐Fc forms retained their selectivity against FGFR1, we performed SPR measurements using purified extracellular domains of FGFR1, PDGFRβ, and VEGFR2. We found that B‐Fc and T‐Fc specifically recognized the extracellular domain of the FGFR1 (Fig. 1C). Furthermore, we found that B‐Fc and T‐Fc were highly specific toward FGFR1 and did not cross‐react with other FGF receptors (Fig. 1D). Using FGFR1 lacking the D1 domain, we demonstrated in SPR experiments that B‐Fc and T‐Fc bound epitopes within the D1 (Fig. 1E). We further validated these data with purified D1 domain of FGFR1 (Fig. 1E).

**Fig. 2:**
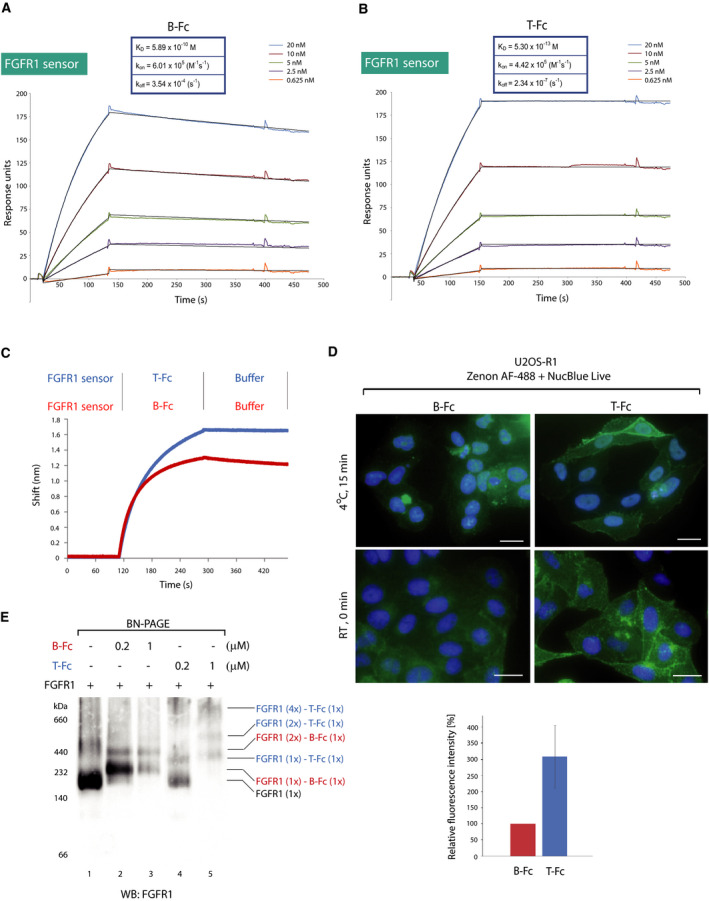
B‐Fc and T‐Fc bind FGFR1 with high affinity. (A, B) SPR‐determined kinetic parameters of the interaction between B‐Fc and T‐Fc, and FGFR1, respectively. The extracellular region of FGFR1 was immobilized on SPR sensors and incubated with various concentrations of B‐Fc and T‐Fc. *K*_D_, *k*_on_, and *k*_off_ values are presented. (C) BLI comparison of B‐Fc and T‐Fc interaction with FGFR1. The extracellular region of FGFR1 was immobilized on BLI sensors and incubated either with B‐Fc or T‐Fc. The association and dissociation profiles were measured. (D) Upper panel, B‐Fc and T‐Fc interaction with FGFR1 on model cells. U2OS‐R1 cells stably producing FGFR1 were incubated with B‐Fc or T‐Fc on ice to prevent internalization of receptor–antibody complexes, or briefly at room temperature. Nuclei were labeled with NucBlue Live; cells were washed, and fixed; and bound antibodies were visualized with Zenon AF‐488 using fluorescence microscopy. Scale bars represent 20 µm. Lower panel, quantification of T‐Fc and B‐Fc cell binding at room temperature performed using zen 2.3 software based on three independent experiments. The signal of B‐Fc was set to 100%, and average intensity of T‐Fc in relation to B‐Fc ±SD was shown. (E) BN‐PAGE analysis of FGFR1 complexes with engineered antibodies. FGFR1‐Fc (0.1 μm) was incubated with B‐Fc (0.2 μm, 1 μm) and T‐Fc (0.2 μm, 1 μm), and proteins were separated on 4–10% BN‐PAGE gels and detected by western blotting.

Next, we determined the kinetic parameters of the interaction between B‐Fc, T‐Fc, and the FGFR1. B‐Fc bound FGFR1 with *K*_D_ = 0.589 nm, which is in agreement with our previous report (Fig. 2A) [25]. T‐Fc displayed *K*_D_ = 0.53 pm, and this increased affinity toward FGFR1 was due to the largely decreased dissociation rate constant (*k*_off_) of T‐Fc, as compared to B‐Fc (Fig. 2B). We confirmed differences in the FGFR1 binding between T‐Fc and B‐Fc using biolayer interferometry (BLI). To this end, the full‐length extracellular domain of FGFR1 was immobilized on BLI sensors and incubated with B‐Fc or T‐Fc. In agreement with SPR data, BLI experiments revealed that T‐Fc displayed much slower dissociation rates than B‐Fc (Fig. 2C). To test the binding of B‐Fc and T‐Fc to the full‐length FGFR1 present in the native environment of the plasma membrane, we used model U2OS‐R1 cells stably producing FGFR1. Equimolar concentrations of B‐Fc and T‐Fc were incubated with U2OS‐R1 cells on ice to prevent receptor‐dependent endocytosis, and then, the cells were washed and cell‐bound engineered antibodies were detected with Zenon AF‐488, a fluorescently labeled Fab fragment recognizing the Fc region of IgG. Alternatively, to retain cell membrane fluidity during binding studies the engineered antibodies were briefly incubated with cells at RT prior to fixation. Fluorescence microscopy experiments confirmed that T‐Fc displayed about three times more efficient binding to the cell surface of FGFR1‐producing cells than B‐Fc (Fig. 2D). Additionally, we confirmed differential cell binding by B‐Fc and T‐Fc using western blotting. Control U2OS cells with minimal FGFR1 expression and U2OS‐R1 cells stably transfected with FGFR1 were incubated with equimolar concentrations of the engineered antibodies. Cells were washed, and bound antibodies were detected with western blotting. T‐Fc displayed significantly more efficient binding to the cell surface FGFR1 in comparison with B‐Fc (Fig. S2).

Next, we determined the impact of B‐Fc and T‐Fc on the oligomeric state of FGFR1. Recombinant FGFR1 was incubated with the engineered antibodies, and the oligomeric state of the receptor was assessed using blue native PAGE (BN‐PAGE). As demonstrated in Fig. 2E, binding of B‐Fc to FGFR1 caused the upshift of FGFR1 on BN‐PAGE gels indicating the formation of B‐Fc‐FGFR1 dimers. In contrast, T‐Fc induced the assembly of high molecular weight complexes of FGFR1, likely receptor tetramers (Fig. 2E). Since B‐Fc and T‐Fc bind the N‐terminal D1 domain of FGFR1, we used BN‐PAGE to analyze changes in the oligomeric state of the D1 domain upon treatment with the engineered antibodies. Whereas B‐Fc mainly induces dimerization of the D1 domain, T‐Fc causes assembly of high molecular weight complexes of the D1 (Fig. S3). We have measured the approximate molecular weight of T‐Fc–FGFR1 complexes using dynamic light scattering (DLS). DLS analyses revealed that the estimated size of T‐Fc–FGFR1 complexes is over 1.1 MDa and might represent receptor tetramers (Fig. S4).

Collectively, these data show that highly specific anti‐FGFR1 engineered tetravalent antibody (T‐Fc) displays improved binding to the extracellular domain of the FGFR1, as compared with its bivalent B‐Fc counterpart. Furthermore, the engineered antibodies differentially regulate the oligomeric state of FGFR1. Whereas the bivalent B‐Fc induces dimerization of FGFR1, the tetravalent T‐Fc triggers clustering of the receptor.

### 3.2 T‐Fc and B‐Fc differentially affect the interaction of FGFR1 with partner proteins

**Fig. 3:**
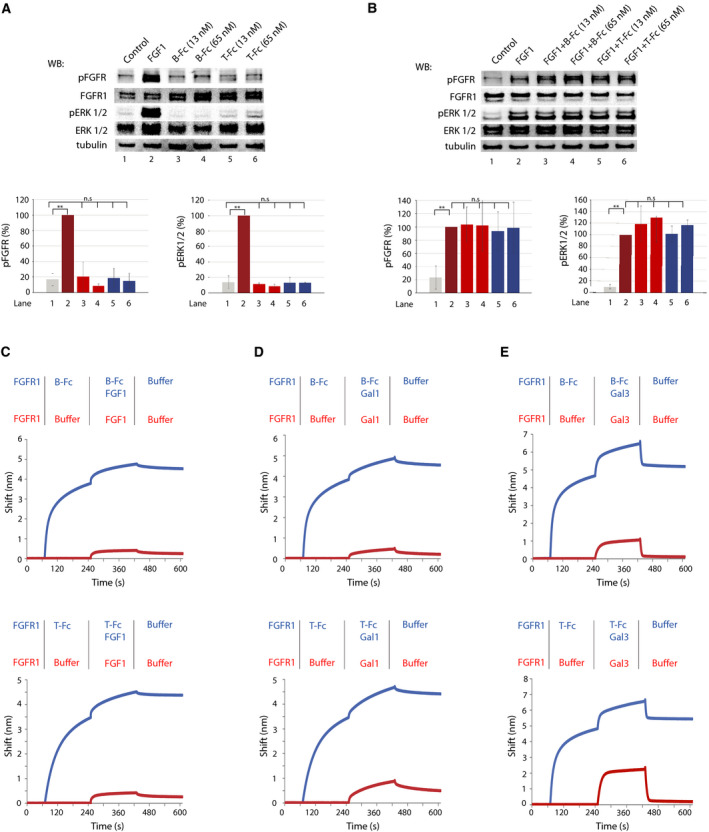
The impact of engineered antibodies on the interaction of FGFR1 with partner proteins. (A) B‐Fc and T‐Fc are unable to activate FGFR1. Serum‐starved NIH3T3 cells were incubated with FGF1 (positive control) or with different concentrations of B‐Fc and T‐Fc. Cells were lysed and activation of FGFR1, and receptor‐downstream signaling was assessed with western blotting (WB). The level of tubulin served as a loading control. Bottom panels: quantification of signaling experiments performed with image lab software. Average values ±SD from at least three independent experiments are shown. The statistical significance was calculated using the *t*‐test; *P < 0.05, **P < 0.005, n.s.—not significant. (B) B‐Fc and T‐Fc have no impact on FGFR1 activation by FGF1. Serum‐starved NIH3T3 cells were incubated with FGF1 alone or in combination with B‐Fc and T‐Fc. Cells were lysed and activation of FGFR1, and receptor‐downstream signaling was assessed with western blotting. The level of tubulin served as a loading control. Bottom panels: quantification of signaling experiments performed with image lab software. Average values ±SD from at least three independent experiments are shown. The statistical significance was calculated using the *t*‐test; *P < 0.05, **P < 0.005, n.s.—not significant. (C) The effect of engineered antibodies on FGF1/FGFR1 interaction. The extracellular domain of FGFR1 was immobilized on BLI sensors and either left untreated or incubated with the saturating concentrations of B‐Fc (left graph) or T‐Fc (right graph). Subsequently, sensors were incubated with FGF1 to assess the impact of antibodies on FGF1/FGFR1 interaction. (D) B‐Fc and T‐Fc have no impact on galectin‐1/FGFR1 interaction. The extracellular domain of FGFR1 was immobilized on BLI sensors and either left untreated or incubated with the saturating concentrations of B‐Fc (left graph) or T‐Fc (right graph). Subsequently, sensors were incubated with galectin‐1 to assess the impact of antibodies on galectin‐1/FGFR1 interaction. (E) T‐Fc partially inhibits binding of galectin‐3 to FGFR1. The extracellular domain of FGFR1 was immobilized on BLI sensors and either left untreated or incubated with the saturating concentrations of B‐Fc (left graph) or T‐Fc (right graph). Subsequently, sensors were incubated with galectin‐3 to assess the impact of antibodies on galectin‐3/FGFR1 interaction.

We next asked whether the interaction of B‐Fc and T‐Fc with FGFR1 results in the receptor activation. For this purpose, NIH3T3 fibroblasts were serum starved and incubated either with FGF1 as a control or with various concentrations of B‐Fc and T‐Fc. Cell lysates were prepared and analyzed with western blotting to assess FGFR1 activation (using antibodies recognizing activated (i.e., tyrosine phosphorylated) FGFR; pFGFR) and the initiation of receptor‐downstream signaling cascades (by monitoring phosphorylated ERK1/2; pERK1/2). Both B‐Fc and T‐Fc were not able to trigger FGFR1‐dependent signaling at tested concentrations (Fig. 3A). Next, we studied whether B‐Fc and T‐Fc had an impact on FGFR1 activation by FGF1. For this purpose, we performed signaling studies using cells preincubated with the excessive concentrations of B‐Fc and T‐Fc prior to the stimulation with FGF1. None of tested engineered antibodies influenced FGFR1 activation by FGF1 (Fig. 3B). In agreement with these findings, BLI analyses revealed that B‐Fc and T‐Fc were not able to block FGF1 binding to the receptor and even slightly improved FGF1‐FGFR1 interaction (Fig. 3C).

Recently, we have reported that galectin family members, galectin‐1 and galectin‐3, interact with the sugar chains of the glycosylated extracellular domain of FGFR1, regulating activity and trafficking of the receptor [31]. Using competitive BLI, we studied the effect of B‐Fc and T‐Fc binding on the receptor interaction with galectin‐1 and galectin‐3. BLI experiments revealed that both B‐Fc and T‐Fc have no impact on the formation of galectin‐1/FGFR1 complexes (Fig. 3D). Interestingly, T‐Fc partially inhibited the interaction of galectin‐3 with FGFR1, while B‐Fc had no effect on galectin‐3/FGFR1 binding (Fig. 3E).

These data suggest that both B‐Fc and T‐Fc have no major impact on FGF1/FGFR1 and galectin‐1/FGFR1 interaction, while larger, tetravalent T‐Fc to some extent blocks the formation of complexes between galectin‐3 and FGFR1.

### 3.3 Clustering of FGFR1 with tetravalent engineered antibody accelerates receptor endocytosis

**Fig. 4:**
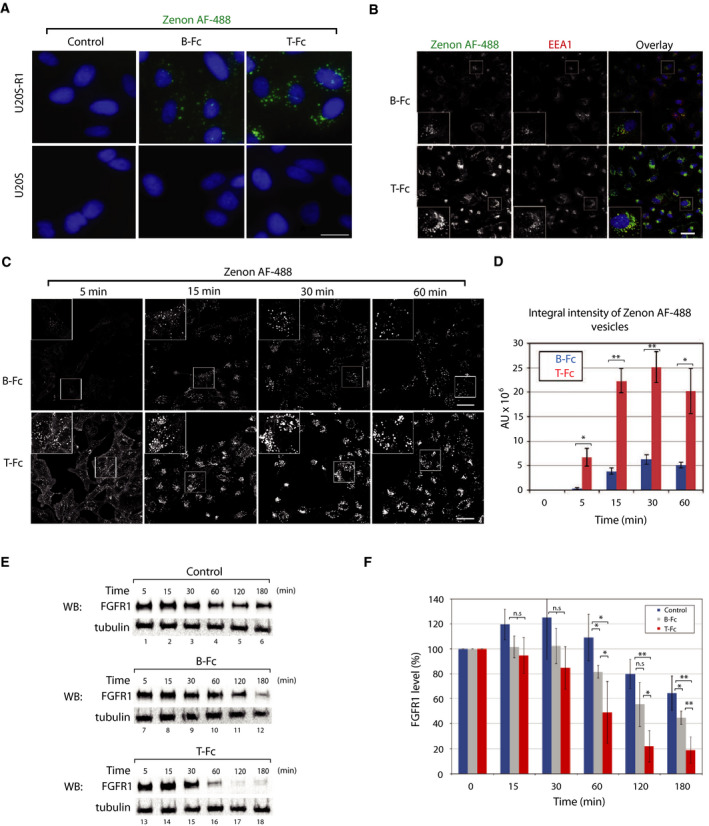
The differential influence of T‐Fc and B‐Fc on FGFR1 internalization. (A) Engineered antibodies are internalized via FGFR1‐dependent endocytosis. U2OS‐R1 cells stably expressing FGFR1 and U2OS cells (control cell line with negligible level of FGFR1) were incubated with 100 nm of B‐Fc and T‐Fc for 30 min. Nuclei were stained with NucBlue Live, cells were fixed, and internalized antibodies were visualized with Zenon AF‐488 using wide‐field fluorescence microscope. Scale bar represents 20 µm. (B) Internalized T‐Fc and B‐Fc are present in endosomes. U2OSR1 cells were incubated with 100 nm B‐Fc and T‐Fc for 15 min, cells were fixed, internalized antibodies were labeled with Zenon AF‐488, and early endosome marker protein EEA1 was detected with immunolabeling. Cells were analyzed with confocal microscopy. Scale bar represents 50 µm. (C) Confocal microscopy analysis of the kinetics of B‐Fc and T‐Fc internalization. U2OSR1 cells were incubated with 100 nm B‐Fc and T‐Fc for different time periods, and internalized antibodies were labeled with Zenon AF‐488 and analyzed with confocal microscopy. Scale bar represents 50 µm. (D) Quantification of B‐Fc and T‐Fc internalization (expressed as integral fluorescence intensity in arbitrary units, AU) using the harmony software. Mean values of three independent experiments of integral intensity of Zenon AF‐488 vesicles ±SEM are shown. *T*‐test was used to assess the statistical significance of measured differences in internalization; *P < 0.05, **P < 0.005. (E) Engineered antibodies induce FGFR1 degradation. U2OS‐R1 cells were serum starved, treated with cycloheximide to inhibit synthesis of new FGFR1 pool, and incubated with equimolar concentrations of B‐Fc and T‐Fc for various time points, or left untreated (control). Cells were lysed, and the level of FGFR1 was determined with western blotting (WB). Tubulin detection was used as an indication of equal loading. Representative results from four independent experiments are shown. (F) Quantitative analyses of FGFR1 degradation (Fig. 4E) upon stimulation with engineered antibodies. FGFR1 band intensities were quantified and corrected for loading differences (intensity of tubulin bands). Average values from four independent experiments ±SD are shown. *T*‐test was used to assess the statistical significance of measured differences in FGFR1 levels,*P < 0.05, **P < 0.005, n.s.—not significant.

In the next step, we studied the cellular uptake of B‐Fc and T‐Fc. To determine the FGFR1 dependence of antibodies’ internalization, we used model U2OS cells with negligible level of FGFR1 and U2OS‐R1 cells stably transfected with FGFR1. Cells were incubated for 30 min with the equimolar concentrations of B‐Fc or T‐Fc, and internalized antibodies were detected with Zenon AF‐488 reagent using fluorescence microscopy. The cellular uptake of B‐Fc and T‐Fc was strictly dependent on the presence of FGFR1 on the cell surface, as parental U2OS cells displayed virtually no intracellular fluorescence of B‐Fc and T‐Fc (Fig. 4A). Interestingly, we observed that the intensity of the intracellular signal of T‐Fc was much higher than that of B‐Fc, which suggested that T‐Fc may display enhanced efficiency of FGFR1‐dependent endocytosis (Fig. 4A). Using confocal microscopy, we confirmed the co‐localization of B‐Fc and T‐Fc with a marker of early endosomes, EEA1 (Fig. 4B). Again, we observed largely increased signal of endosome‐localized T‐Fc as compared with B‐Fc (Fig. 4B).

To determine the efficiency of B‐Fc and T‐Fc internalization, we applied confocal microscopy and quantitative image analysis with the harmony software. The engineered antibodies were incubated in equimolar concentrations with U2OS‐R1 cells for different time periods, and the integral intensity of intracellular Zenon AF‐488 fluorescence was measured. The quantitative analyses of the uptake of the engineered antibodies revealed that the tetravalent T‐Fc is endocytosed with about five times higher efficiency than the bivalent B‐Fc (Fig. 4C,D).

Since the internalization of B‐Fc and T‐Fc strictly depends on FGFR1, we studied the effect of differential efficiencies of B‐Fc and T‐Fc cellular uptake on FGFR1 trafficking. Upon internalization, FGFR1‐ligand complexes are mainly targeted to lysosomes for degradation [36]. We employed a biochemical assay where we blocked the synthesis of new FGFR1 molecules with cycloheximide and analyzed with western blotting the levels of FGFR1 in time upon stimulation with B‐Fc and T‐Fc. The time‐dependent decrease in FGFR1 level is attributed to the lysosomal degradation of the internalized receptor [5,6]. In nontreated cells within the time of the experiment, we observed very slight reduction in FGFR1 level that likely corresponds to the constitutive, ligand‐independent receptor endocytosis (Fig. 4E,F). Incubation of cells with B‐Fc significantly accelerated FGFR1 degradation, especially in the later time points (Fig. 4E,F). In agreement with microscopy studies, T‐Fc largely enhanced FGFR1 degradation, as compared to the untreated control and cells stimulated with B‐Fc (Fig. 4E,F).

Taken together, these data demonstrate that B‐Fc and T‐Fc are internalized by FGFR1‐dependent endocytosis. Importantly, our results imply that the clustering of FGFR1 with T‐Fc largely enhances the cellular uptake of FGFR1.

### 3.4 Clustering of FGFR1 with T‐Fc alters the mechanism of receptor internalization

**Fig. 5:**
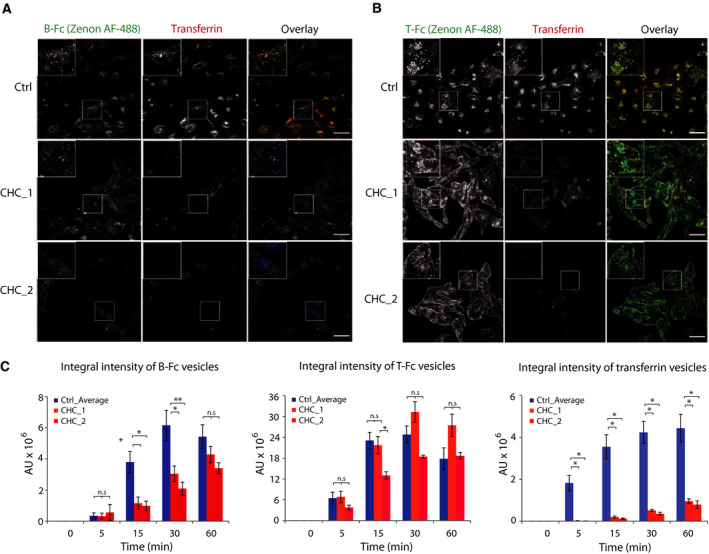
T‐Fc is internalized via clathrin‐independent endocytosis. U2OS‐R1 cells were subjected to siRNA‐mediated silencing of clathrin heavy chain (CHC) to inhibit clathrin‐mediated endocytosis. Two different siRNA against CHC were used. Cells were incubated with 100 nm B‐Fc (A) and T‐Fc (B), and fluorescently labeled transferrin as an internal control of CME inhibition for the indicated time periods. Internalized engineered antibodies were visualized with Zenon AF‐488 using confocal microscopy. Scale bars represent 50 µm. (C) Quantification of the effects of CME inhibition on the uptake of B‐Fc, T‐Fc, and transferrin. Internalization of B‐Fc, T‐Fc, and transferrin‐containing vesicles (expressed as integral fluorescence intensity in arbitrary units, AU) was measured with the harmony software. Average values of three independent experiments ±SEM. *T*‐test was used to assess the statistical significance of measured differences; *P < 0.05, **P < 0.005, n.s.—not significant.

Since we observed largely enhanced efficiency of T‐Fc uptake in relation to that of B‐Fc, we wondered whether the engineered antibodies utilize the same endocytic route with different efficiencies or engage distinct internalization pathways. It was reported that FGF1/FGFR1 dimeric complexes are internalized via clathrin‐mediated endocytosis (CME) [30,35, 36, 37]. We have recently demonstrated that the dimerization of FGFR1 with bivalent antibody triggers CME of FGFR1/antibody complexes [5,6]. We applied siRNA‐mediated clathrin heavy chain (CHC) silencing in conjunction with quantitative confocal microscopy to study the contribution of CME to the cellular uptake of FGFR1/B‐Fc and FGFR1/T‐Fc complexes. The effectiveness of CHC downregulation was confirmed with western blotting (Fig. S5). Additionally, we used a fluorescently labeled transferrin, an established CME cargo as an internal control to monitor CME inhibition [47]. The CHC knockdown largely blocked the internalization of transferrin, confirming the efficient inhibition of CME (Fig. 5A–C). The cellular uptake of B‐Fc was largely decreased upon CME inhibition till 30 min after antibody addition (Fig. 5A,C). However, after 60 min we observed a compensatory mechanism of B‐Fc internalization in the absence of clathrin (Fig. 5C). We also observed that the efficiency of T‐Fc endocytosis was unaffected by CME block (Fig. 5B,C).

These data suggest that clustering of FGFR1 with tetravalent T‐Fc modifies the mechanism of the receptor internalization. While FGFR1 dimers predominantly utilize CME, the cell uptake of larger oligomeric structures of the receptor formed by T‐Fc is split between two pathways: clathrin‐mediated and clathrin‐independent endocytosis (CIE). In addition, it seems that the perturbation of B‐Fc uptake in the absence of clathrin can be compensated by an endocytic pathway operating with slower kinetics than CME.

### 3.5 Dynamin‐dependent, clathrin‐independent endocytic pathways contribute to the internalization of T‐Fc/FGFR1 complexes

**Fig. 6:**
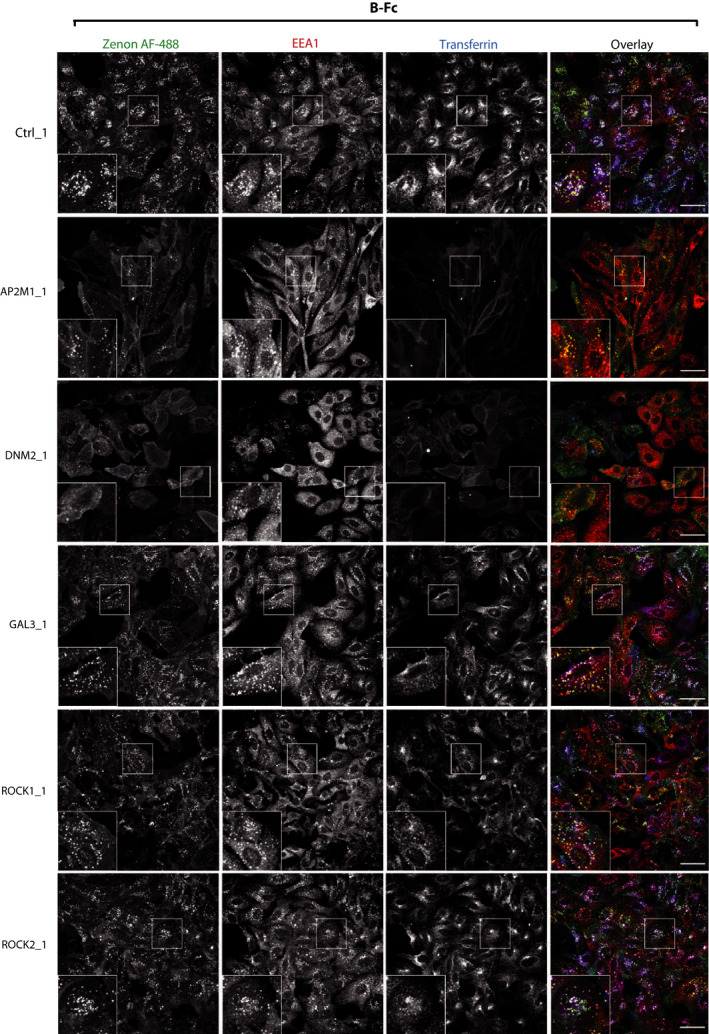
The effect of inhibition of clathrin‐independent endocytic routes on the cellular uptake of B‐Fc. U2OS‐R1 cells were subjected to siRNA‐mediated silencing of a μ2 subunit of the AP2 complex (AP2μ2), dynamin‐2 (DNM2), galectin‐3 (GAL3), ROCK1, and ROCK2. Cells were incubated for 15 min with 100 nm B‐Fc and fluorescently labeled transferrin as an internal control of CME inhibition. Internalized B‐Fc was visualized with Zenon AF‐488, and its co‐localization with immunolabeled EEA1 was determined with confocal microscopy. Scale bars represent 50 µm.

**Fig. 7:**
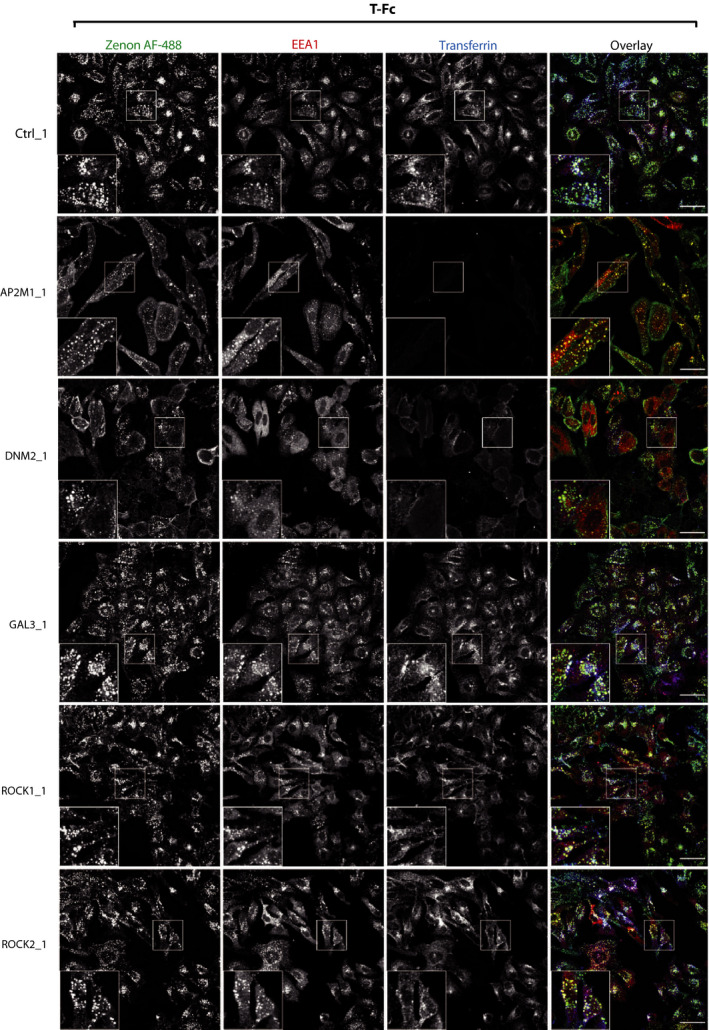
The effect of inhibition of clathrin‐independent endocytic routes on the cellular uptake of T‐Fc. U2OS‐R1 cells were subjected to siRNA‐mediated silencing of a μ2 subunit of the AP2 complex (AP2μ2), dynamin‐2 (DNM2), galectin‐3 (GAL3), ROCK1, and ROCK2. Cells were incubated for 15 min with 100 nm T‐Fc and fluorescently labeled transferrin as an internal control of CME inhibition. Internalized T‐Fc was visualized with Zenon AF‐488, and its co‐localization with immunolabeled EEA1 was determined with confocal microscopy. Scale bars represent 50 µm.

**Fig. 8:**
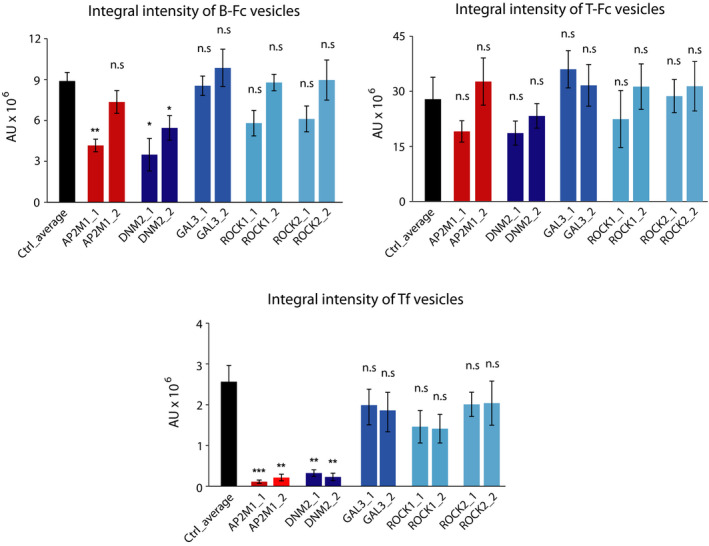
Quantitative analyses of B‐Fc and T‐Fc internalization upon inhibition of clathrin‐independent endocytic pathways. Quantification of engineered antibodies internalization. Cells were treated as in Figs 6 and 7, Fig. S1 and S2, and the efficiency of B‐Fc, T‐Fc, and transferrin internalization (expressed as integral fluorescence intensity in arbitrary units, AU) was assessed with the harmony software. Average values from three independent experiments ±SEM are shown. T‐test was used to assess the statistical significance of measured differences,*P < 0.05, **P < 0.005, n.s.—not significant.

The CIE includes several distinct internalization pathways [51, 52, 53]. To study which CIE route mediates the endocytosis of T‐Fc, we employed siRNA‐mediated knockdown of key CIE‐specific proteins. We downregulated expression of dynamin‐2, which is involved in CME and in several CIE routes, galectin‐3, FGFR1‐binding protein mediating the clathrin‐independent carriers (CLIC) pathway, and ROCK1 and ROCK2 proteins involved in RhoA‐dependent CIE [31,51]. As a control, we knocked down a μ2 subunit of the adaptin‐2 complex (AP2μ2), an CME adaptor [53,54]. The effectiveness of siRNA‐mediated downregulation of endocytic proteins was confirmed with western blotting (Fig. S5). B‐Fc and T‐Fc were incubated with siRNA‐treated U2OS‐R1 cells, and endocytosed engineered antibodies were visualized with Zenon AF‐488. Additionally, we supplemented the cells with fluorescently labeled transferrin to monitor the selectivity of endocytic routes inhibition. The efficiency of engineered antibodies internalization and their co‐localization with EEA1 and transferrin were assessed with high‐content quantitative confocal microscopy. In agreement with previous findings, the downregulation of AP2μ2 or dynamin‐2 largely inhibited the uptake of B‐Fc and transferrin (Fig. 6, Fig. S6). The silencing of galectin‐3, ROCK1 or ROCK2 had no impact on the internalization of B‐Fc, confirming the exclusive involvement of CME in the uptake of FGFR1/B‐Fc complexes (Fig. 6, Fig. S6). Strikingly, the single knockdown of all studied proteins of CIE and CME had no significant influence on the endocytosis of T‐Fc (Fig. 7, Fig. S7). These data were confirmed when imaging data were quantitatively analyzed (Fig. 8). These results suggested that T‐Fc‐FGFR1 complexes might simultaneously utilize several distinct endocytic routes to enter the cells.

**Fig. 9:**
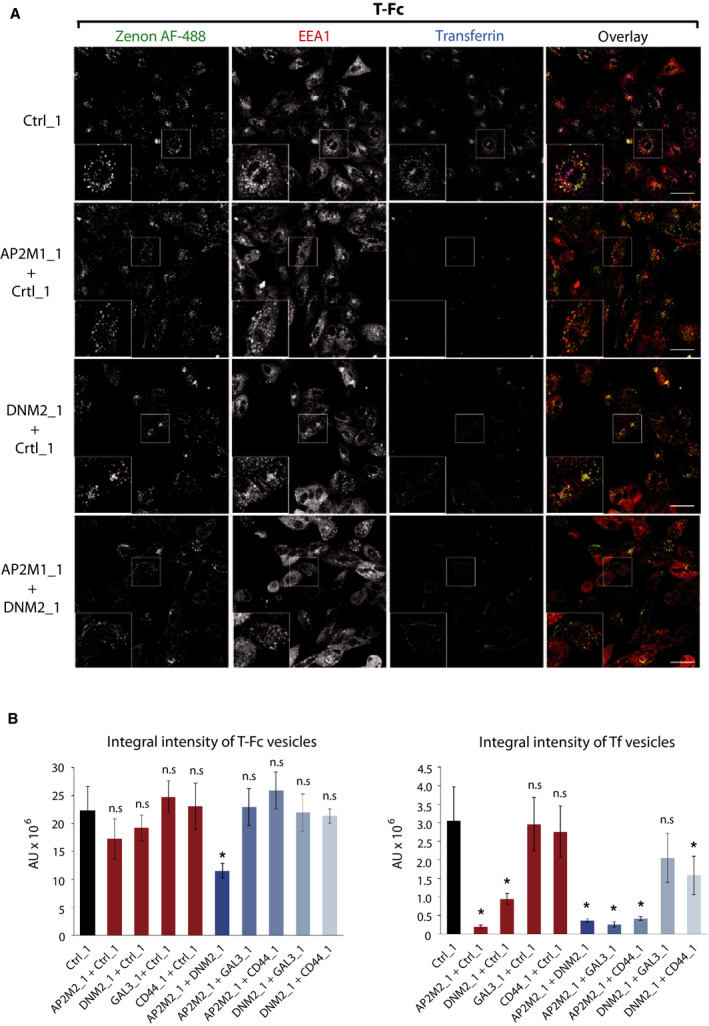
Different endocytic routes mediate cellular uptake of T‐Fc. (A) Representative confocal microscopy images of U2OS‐R1 cells showing the effect of simultaneous depletion of AP2µ2 (AP2M1) and dynamin‐2 (DNM2) on the internalization of T‐Fc. Cells were incubated for 60 min with 100 nm T‐Fc and fluorescently labeled transferrin as an internal control of CME inhibition. Internalized T‐Fc was visualized with Zenon AF‐488, and its co‐localization with immunolabeled EEA1 was determined using confocal microscopy. Scale bars represent 50 µm. (B) Quantification of internalization of the engineered antibodies upon double‐depletion of different endocytic proteins. The efficiency of T‐Fc and transferrin internalization (expressed as integral fluorescence intensity in arbitrary units, AU) was assessed with harmony software. Average values from three independent experiments ±SEM are shown. T‐test was used to assess the statistical significance of measured differences; *P < 0.05, **P < 0.005, n.s.—not significant.

We further searched for endocytic pathways that mediate T‐Fc uptake by simultaneously knocking down distinct CME and CIE components. Among the tested combinations only the concurrent depletion of AP2μ2 and dynamin‐2 significantly blocked the internalization of T‐Fc (Fig. 9A,B). Simultaneous silencing of AP2μ2 and proteins involved in dynamin‐independent CIE pathways, galectin‐3 and CD44 had no effect on T‐Fc endocytosis (Fig. 9B).

Our results imply that FGFR1 clustering with tetravalent T‐Fc alters the mechanism of the receptor internalization. The uptake of FGFR1–T‐Fc complexes is split between two distinct endocytic pathways: CME and dynamin‐dependent CIE. The depletion of single CME or CIE components is not sufficient to block T‐Fc uptake, as it is compensated by remaining active endocytic routes. In contrast, B‐Fc follows CME, a typical pathway for FGFR1 dimers.

## 4 Discussion

**Fig. 10:**
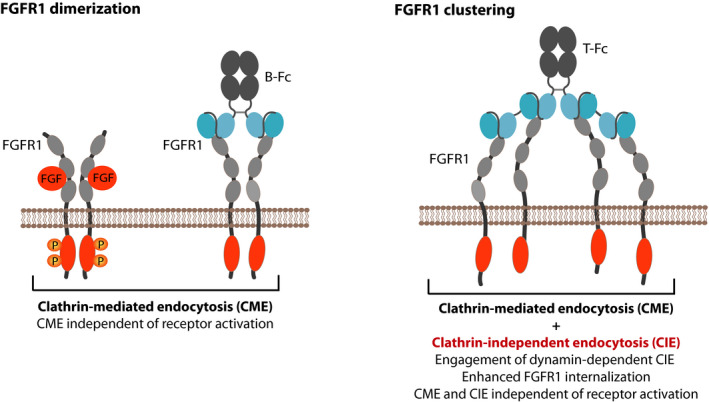
Hypothetical model of the effect of differential FGFR1 clustering on the receptor endocytosis. FGFR1 dimerization, either with FGF1 or bivalent antibodies, like B‐Fc, induces CME of the receptor. CME initiation is independent of FGFR1 activation. Clustering of FGFR1 into large structures on the plasma membrane with tetravalent T‐Fc largely improves the cellular uptake of FGFR1–antibody complexes. Furthermore, FGFR1 clustering changes the mechanism of the receptor endocytosis by engaging dynamin‐dependent CIE pathways. Similarly to CME, CIE of FGFR1 does not require receptor activation.

The cellular trafficking of cell surface receptors, including RTKs, constitutes a mean for spatiotemporal adjustment of cellular signaling [55]. The aberrant RTKs transport results in enhanced propagation of signals, often observed in cancer cells [56]. Although the mechanism of FGFR1 activation and signaling is well studied, the knowledge about trafficking of FGFR1 is far from complete. Recent proteomic studies revealed several proteins that may modulate intracellular transport of FGFR1, however, the exact role of these factors in FGFR1 trafficking awaits further studies [31,42]. FGFR1 is subjected to the constitutive low‐rate internalization from the plasma membrane. Binding of FGFs induces FGFR1 dimerization and autoactivation and stimulates cellular uptake of FGFR1, which occurs via CME [30,37]. We have recently demonstrated that CME of FGFR1 is independent of receptor tyrosine kinase activity and is solely triggered by changes in the oligomeric state of the receptor in the plasma membrane [5,6]. Dimerization of FGFR1 either by FGF1 or engineered bivalent antibodies stimulates the uptake of FGFR1 via CME (Fig. 10) [5,6]. Data presented in this report extend these findings and show that the cellular trafficking of FGFR1 is, in general, dictated by the spatial organization of FGFR1 in the plasma membrane. Clustering of FGFR1 with engineered tetravalent antibody largely improves the cellular uptake of FGFR1. The fivefold increase in the efficiency of FGFR1 internalization is achieved by changes in the employed mechanism of endocytosis. Our data suggest that, in contrast to FGFR1 dimers internalized predominantly by CME, the cell entry of larger FGFR1 oligomers induced by T‐Fc is split between two pathways: CME and dynamin‐dependent CIE (Fig. 10). In agreement with our findings, the CIE of FGFR1 was previously reported [14,34,45].

Similarly to CME, the highly efficient CIE of FGFR1 clusters occurs without the receptor activation. Our data suggest that the organization of FGFR1 in the plasma membrane and not receptor phosphorylation is sensed by distinct intracellular endocytic machineries involved in CME or CIE. Engineered antibodies used in this study do not bind the ligand recognition pocket formed by D2 and D3 domains of FGFR1 and therefore are unable to activate the receptor. The slightly enhanced FGF1/FGFR1 interaction in the presence of engineered antibodies might be due to the restriction of the D1 domain autoinhibitory function. The D1 is involved in the intramolecular interaction with D2 and D3 domains of FGFR1, shielding the binding site for FGFs [4]. Binding of the D1 by engineered antibodies may block this intramolecular interaction, facilitating the access of FGF1 to the ligand recognition region within FGFR1.

We have recently uncovered the interplay between extracellular galectins and FGFR1 in the adjustment of receptor‐dependent signaling and trafficking [31]. Interestingly, our binding studies suggest that engineered tetravalent antibody may inhibit galectin‐3/FGFR1 interaction. Since smaller, bivalent antibody is unable to block formation of galectin‐3/FGFR1 complexes, the reason of the observed inhibitory activity of larger tetravalent antibody is likely to be due to the steric nature. Galectin‐3 plays numerous important cellular functions and is implicated in cancer [57,58]. Furthermore, galectin‐3 is involved in CIE of several cargos [51,59]. The physiological significance of galectin‐3/FGFR1 interplay is currently unknown,however, the high‐affinity tetravalent antibody described in this study can be used to partially uncouple galectin‐3/FGFR1 interaction, which may be of potential relevance for therapeutic purposes in cancer treatment.

The major goal of targeted cancer treatment with ADCs is to reduce unwanted side effects by highly efficient and selective delivery of drugs into cancer cells and avoiding the healthy ones. The selective recognition of cancer‐specific antigen followed by receptor‐mediated cellular uptake of ADCs constitutes the major factor behind ADCs specificity. Here, we report that clustering of FGFR1 with tetravalent antibody largely improves the internalization of receptor–antibody complexes and directs these proteins for lysosomal degradation. Tetravalent antibody‐mediated FGFR1 clustering largely improves the uptake efficiency, which may boost the selective delivery of drugs into FGFR1‐overproducing cancer cells. This may be partially due to the very high affinity of tetravalent antibody for FGFR1, a factor recently demonstrated by us to play a significant role in the effectiveness of anti‐FGFR1 antibody internalization [49]. The activity of distinct endocytic pathways can be altered depending on physiological conditions [60, 61, 62]. Importantly, constantly dividing cancer cells may downregulate CME and thus increase the lifetime of activated RTKs on the cell surface, promoting oncogenic signaling [63]. Typical bivalent anti‐FGFR1 antibodies used in ADCs utilize CME for the cell entry, and thus, their uptake by cancer cells with downregulated CME might be limited. FGFR1 clustering with T‐Fc activates CIE pathways, ensuring efficient intracellular delivery of antibody to the FGFR1‐overproducing cells even upon CME inhibition or disturbance. Furthermore, T‐Fc is efficiently taken up by the cells even when particular CIE pathways are inhibited, suggesting that alterations in protein trafficking displayed by cancer cells will have a very limited impact on T‐Fc internalization.

## 5 Conclusions

We demonstrate the significance of FGFR1 spatial distribution for the receptor trafficking. Our data, besides fundamental importance for FGFR1 biology, might be relevant for the design of highly efficient targeting molecules for ADC therapy of FGFR1‐overproducing cancers.

## Conflict of interest

The authors declare no conflict of interest.

## Author contributions

ŁO and JO designed and supervised the project; MP, KJ, AS‐W, MAK, MZ, MM, and ŁO designed the experiments; MP, KJ, AS‐W, NP, MAK, JS, and ŁO performed the experiments; all authors analyzed data; MP, KJ, and ŁO prepared the figures; and ŁO and MP wrote first draft of the manuscript. All authors discussed results of the experiments, edited, and approved final version of the manuscript.

## Supporting information


**Fig. S1.** Mass spectrometry analysis of purified T‐Fc.
**Fig. S2.** Interaction of B‐Fc and T‐Fc with cells.
**Fig. S3.** BN‐PAGE analyses of the FGFR1 D1 domain complexes with engineered antibodies.
**Fig. S4.** Dynamic light scattering analyses of T‐Fc – FGFR1 complexes.
**Fig. S5.** Western blotting analysis of the efficiency of siRNA‐mediated knockdown.
**Fig. S6.** The effect of inhibition of clathrin‐ dependent and ‐independent endocytic routes on the cellular uptake of B‐Fc.
**Fig. S7.** The effect of inhibition of clathrin‐ dependent and ‐independent endocytic routes on the cellular uptake of T‐Fc.
