# Supplementary material for: FGFR1 clustering with engineered tetravalent antibody improves the efficiency and modifies the mechanism of receptor internalization
Source: Mol Oncol. 2020 Jul 3;14(9):1998–2021. doi: 10.1002/1878-0261.12740 (PMC7463352; doi:10.1002/1878-0261.12740)
Supplement: Supplementary file 1 — Fig. S1. Mass spectrometry analysis of purified T‐Fc. Fig. S2. Interaction of B‐Fc and T‐Fc with cells. Fig. S3. BN‐PAGE analyses of the FGFR1 D1 domain complexes with engineered antibodies. Fig. S4. Dynamic light scattering analyses of T‐Fc – FGFR1 complexes. Fig. S5. Western blotting analysis of the efficiency of siRNA‐mediated knockdown. Fig. S6. The effect of inhibition of clathrin‐ dependent and ‐independent endocytic routes on the cellular uptake of B‐Fc. Fig. S7. The effect of inhibition of clathrin‐ dependent and ‐independent endocytic routes on the cellular uptake of T‐Fc. [file MOL2-14-1998-s001.docx]

**­Supplemental Information**

**FGFR1 clustering with engineered tetravalent antibody improves the efficiency and modifies the mechanism of receptor internalization**

Marta Pozniak^1^, Aleksandra Sokolowska-Wedzina^1#^, Kamil Jastrzebski^2#^, Jakub Szymczyk^1^, Natalia Porebska^1^, Mateusz Adam Krzyscik^1, 3^, Malgorzata Zakrzewska^1^, Marta Miaczynska^2^, Jacek Otlewski^1^ and Lukasz Opalinski^1^*

**Supplementary Figures Legends**

**Figure S1. Mass spectrometry analysis of purified T-Fc.** The molecular mass of T-Fc was assessed by MALDI-TOF/TOF MS (Applied Biosystem AB 4800+) using sinapic acid as a matrix. The theoretical Mw of the proteinaceous core of T-Fc is 76684,95 Da (T-Fc is glycosylated in the Fc region).

**Figure S2. Interaction of B-Fc and T-Fc with cells.** U2OS and U2OS-R1 cells were incubated briefly at RT with engineered antibodies (100 nM), washed and lysed. Cell-bound engineered antibodies were detected with western blotting using anti-Fc antibodies. The quantification of signals was performed with Image Lab Software from three independent experiments. The statistical significance was assessed using t-test; * p<0.05, **p<0.005, n.s. - not significant.

**Figure S3. BN-PAGE analyses of the FGFR1 D1 domain complexes with engineered antibodies**. B-Fc (1 μM), T-Fc (1 μM) were mixed with D1-GST (0.165 μM) and incubated at RT for 10 min. Proteins were separated on 4-10% BN-PAGE gels and analyzed with western blotting using anti-GST antibodies.

**Figure S4. Dynamic light scattering analyses of T-Fc – FGFR1 complexes.** T-Fc (0.2 mg/ml) (**A**), FGFR1-Fc (0.2 mg/ml) (**B**) and their mixture (FGFR1-Fc (0.15 mg/ml), T-Fc (0.3 mg/ml) (**C**) were subjected to DLS measurements. DLS data was collected and analyzed using DYNAMICS V7 software (Wyatt Technology, CA). DLS-based hydrodynamic diameters and molecular mass were determined by cumulants analysis using Rayleigh Spheres model.

**Figure S5. Western blotting analysis of the efficiency of siRNA-mediated knock-down.** U2OS-R1 cells treated with control siRNA or siRNA targeting distinct endocytic proteins were lysed and subjected to western blotting.

**Figure S6. The effect of inhibition of clathrin- dependent and -independent endocytic routes on the cellular uptake of B-Fc.** U2OS-R1 cells were subjected to siRNA-mediated silencing of a μ2 subunit of the AP2 complex (AP2μ2), dynamin-2 (DNM2), galectin-3 (GAL3), ROCK1 and ROCK2. The second set of independent siRNAs against endocytic proteins was used as in experiments shown in Fig. 6. Scale bars represent 50 µm.

**Figure S7. The effect of inhibition of clathrin- dependent and -independent endocytic routes on the cellular uptake of T-Fc.** U2OS-R1 cells were subjected to siRNA-mediated silencing of a μ2 subunit of the AP2 complex (AP2μ2), dynamin-2 (DNM2), galectin-3 (GAL3), ROCK1 and ROCK2. The second set of independent siRNAs against endocytic proteins was used as in experiments shown in Fig. 7. Scale bars represent 50 µm.

**Supplementary Figures**

**
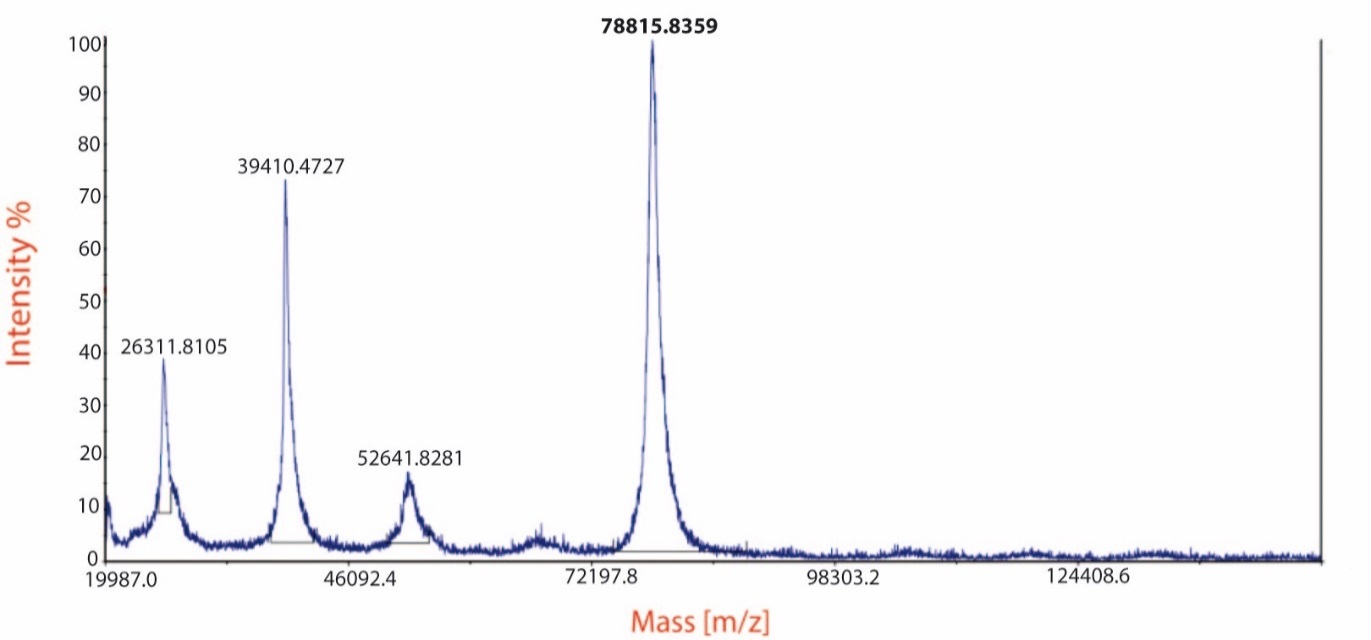
**

**Figure S1**

**
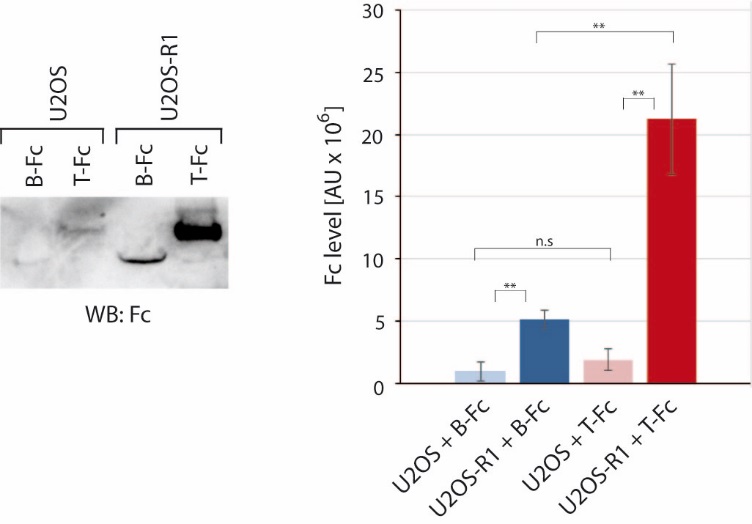
**

**Figure S2**

**
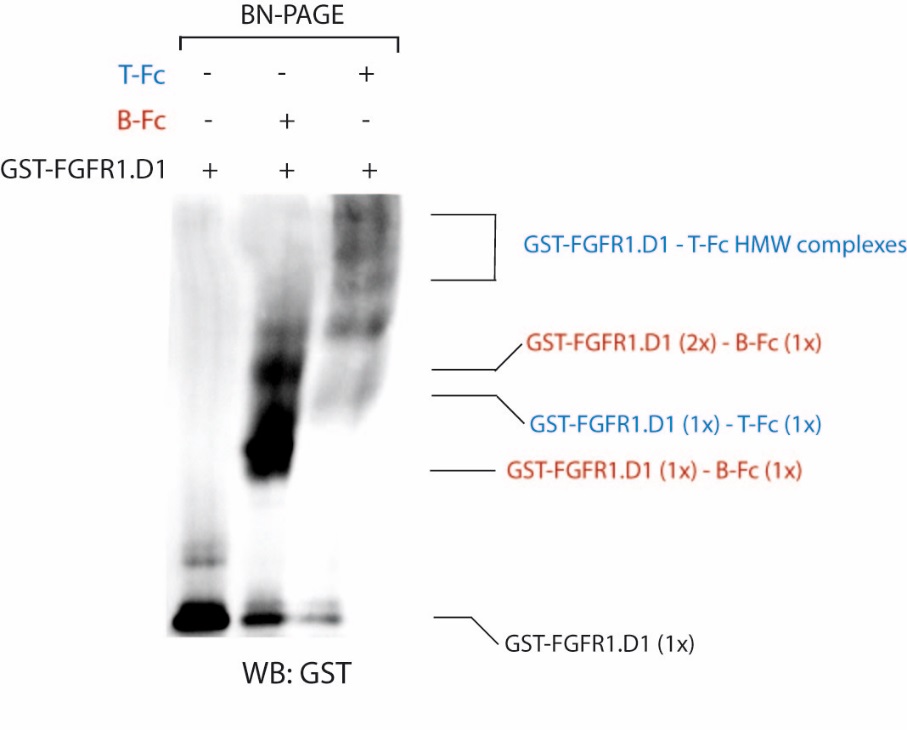
**

**Figure S3**

**
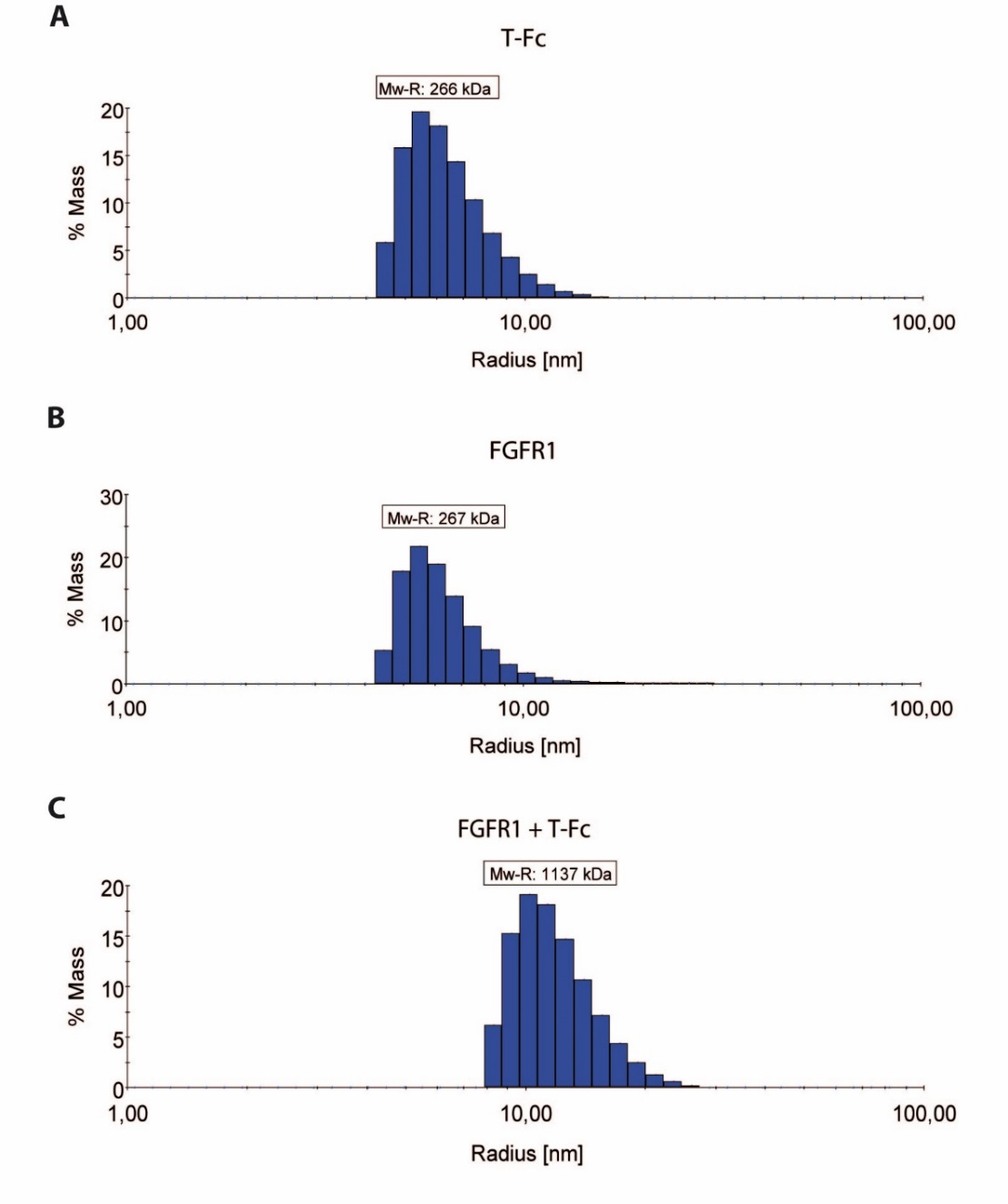
**

**Figure S4**

**
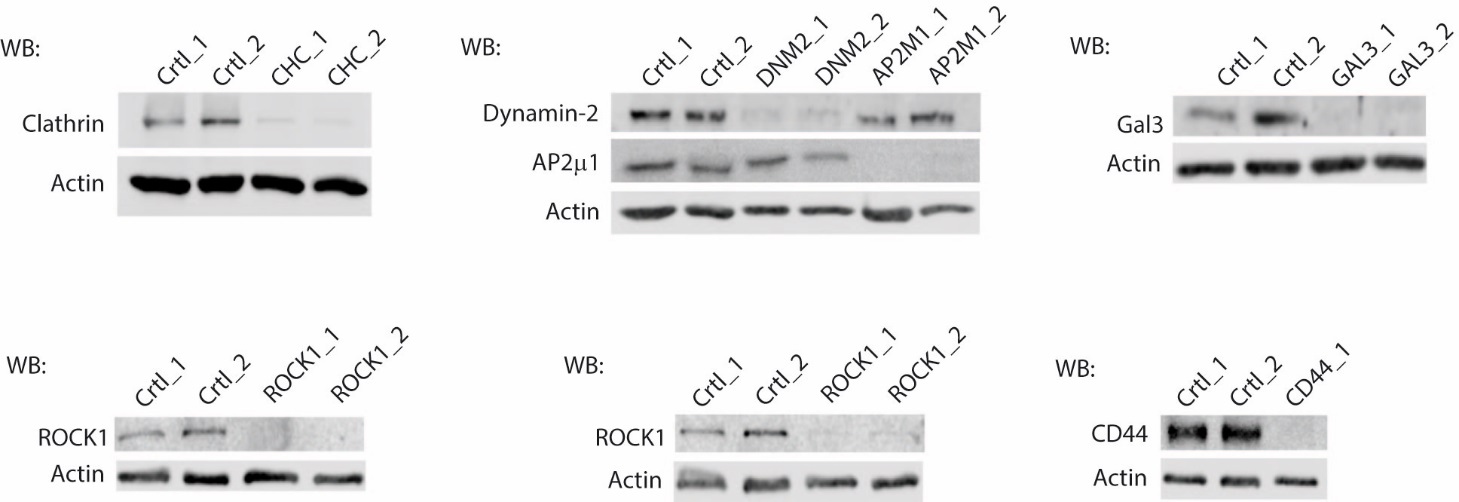
**

**Figure S5**


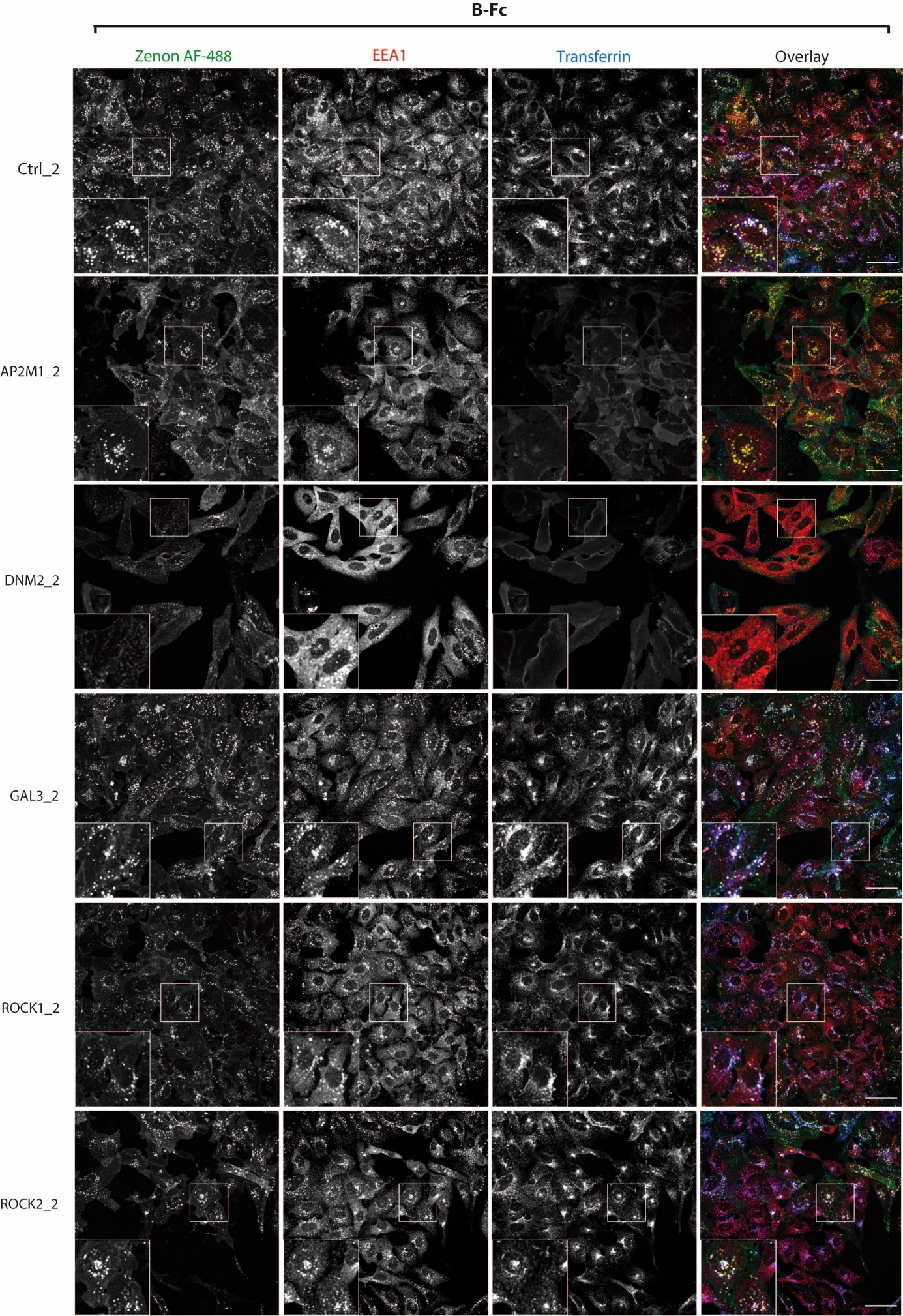


**Figure S6**

**
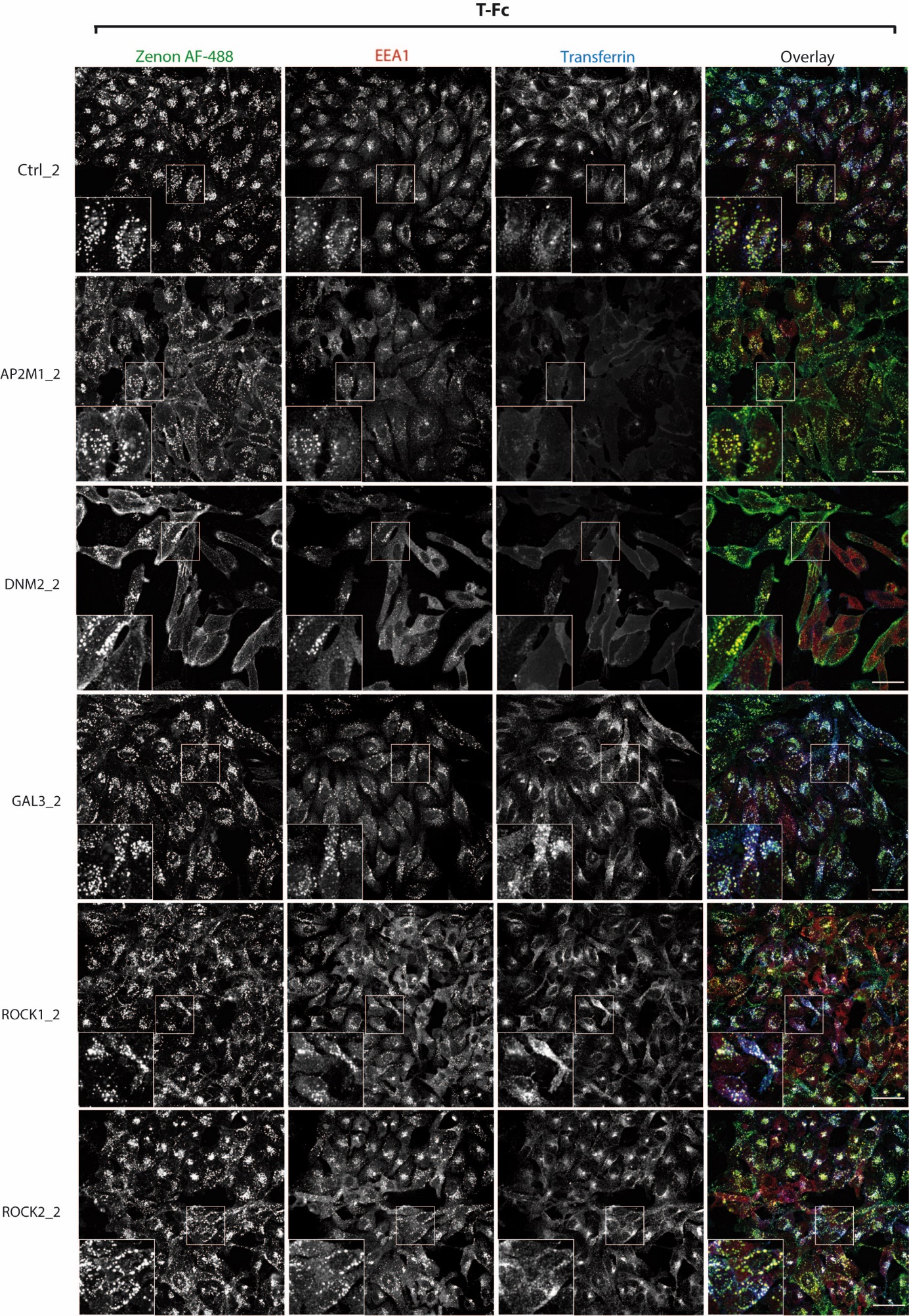
**

**Figure S7**
